# Toxicological Effects of Cypermethrin on Female Albino Rats

**DOI:** 10.4103/0971-6580.75844

**Published:** 2011

**Authors:** G. K. Sangha, Kamalpreet Kaur, K. S. Khera, Balwinder Singh

**Affiliations:** Department of Zoology, Punjab Agricultural University, Ludhiana-141004, India; 1 Department of Entomology, Punjab Agricultural University, Ludhiana-141004, India

**Keywords:** Cypermethrin, endocrine glands, female rats, heart, kidney, liver, lungs, spleen

## Abstract

A study was undertaken to evaluate the effects of cypermethrin on reproduction of female albino rats. The experimental rats were fed cypermethrin at 50 mg/kg b. wt. continuously for a period of 2 and 4 weeks. Feed and water intake was also noted daily for control, vehicle treated and cypermethrin-treated rats. It was observed that there was no effect on feed and water intake in treated rats as compared to the control group. Chronic exposure to cypermethrin for 4 weeks resulted in loose fecal pellets and hyperirritability in the treated rats. Treatment related mortality also occurred at the 4^th^ wk of treatment. Significant changes in body weight and various organ weights due to cypermethrin were observed along with disruption of estrous cycle in rats. The body weight gain in treated rats was lower at both 2 and 4 weeks as compared to the control rats. The weight of liver and spleen decreased, while that of kidneys increased as compared to the control rats. Thyroid and adrenal showed increase in weight at both 2 and 4 weeks of treatments.

## INTRODUCTION

Synthetic pyrethroid insecticides are now being substituted for pest control and increased production.[1] They are most widely used group of pesticides due to their low toxicity to mammals, birds and insects.[2] Cypermethrin, a composite pyrethroid, is a broad-spectrum insecticide and fast acting neurotoxin with good contact and stomach action.[3] Consistent with its lipophilic nature, it has been found to accumulate in body fat, skin, liver, kidneys, adrenal glands, ovaries and brain.[3] Cypermethrin and dichlorodiphenyltrichloroethane (DDT) have been detected in human milk from malarial endemic areas in South Africa.[4] There have been extensive histomorphological, toxicological and biochemical reports on cypermethrin toxicity in different species of animals.[5,6] However, no information is available about the effect of cypermethrin in female rats. So, the present study was conducted to elucidate the toxicity of cypermethrin at low dose levels in female albino rats.

## MATERIALS AND METHODS

Sexually mature female albino rats of 3 months age, weighing 110-150 g, were obtained from Guru Angad Dev Veterinary and Animal Sciences University (GADVASU), Ludhiana. The animals were housed in groups of two rats per cage. The rats were acclimatized for 10 days before using them for experimentation. Their ovarian cyclicity was checked by daily microscopic examination of vaginal smears. Rats showing a 4-day estrous cycle were selected for the present investigation. Commercial formulation of cypermethrin (Rallis India Limited, Secunderabad, Hyderabad) having 25% Emulsifiable Concentrate (EC) was used for the present study. Adequate dilutions were made with vegetable oil to achieve the test concentration of 50 mg/kg. The test concentration of cypermethrin was calculated from the percentage of active ingredient of commercial formulation of cypermethrin. Rats were intubated orally with cypermethrin at 50 mg/kg b. wt. daily for 2 and 4 weeks. Group of rats receiving similar amount of vegetable oil and distilled water served as control rats. All the animals (control and cypermethrin treated) were observed daily for clinical symptoms like salivation, activity, irritability, fecal pellet conditions, diarrhea, eyeball movement, weakness, coarse tremor, paralysis of limb, wounds and mortality, etc. Feed and water intake was also noted for control and cypermethrin-treated rats. Cyclicity was checked daily for the control and treated rats throughout the experimentation period. The body weight of the rats was taken before the start of the treatment in control as well as in cypermethrin treatment groups. During the duration of experiment, the rats were weighed weekly to determine the change in body weight. On completion of the experiment, the rats were sacrificed and various organs like liver, spleen, heart, lungs, kidney and endocrine glands were excised and weighed.

## RESULTS AND DISCUSSION

### General toxicity symptoms

In the first and second weeks of treatment, no morphological or toxicological effect of pesticide was observed. However, the chronic exposure of cypermethrin for 4 weeks resulted in loose fecal pellets and hyperirritability in treated rats. Various studies have investigated the toxic effects of cypermethrin in mammals, revealing increase in salivation, lack of coordination, muscle tremor and convulsions.[7] These signs of toxicity indicate that the target for this compound is the central nervous system in mammals.[7,8]

It was observed that there was no effect on feed and water intake in treated rats as compared to control groups [Table 1]. Rats exposed to liquid mosquito repellent (LMR) containing allethrin (3.6% w/w) showed no significant effect on food consumption.[2] Lambda cyhalothrin also had no significant effect on water intake.[9] However, cypermethrin when given to white rabbits showed reduced feed and water intake in treated animals as compared to control groups.[10] The estrous cycle was slightly disturbed in all the treated rats. Persistent estrous was observed in some of treated rats at third week of treatment as compared to control groups. Mortality also occurred on 17^th^ and 24^th^ days of treatment in two rats treated with cypermethrin, while all the control rats remained healthy throughout the experimentation. Treatment-related deaths (two with low dose and five with high dose) have also been observed in lambda cyhalothrin treated rats.[9]

**Table 1:** Effect of cypermethrin on feed and water intake in control and treated female albino rats after 2 and 4 weeks of treatment

Components	After 2 weeks			After 4 weeks		
	Control I	Control II	Treated	Control I	Control II	Treated
Feed intake (g/100 g b. wt.)	9.25±0.08	8.23±0.10	8.60±0.01	9.72±0.07	8.43±0.01	8.50±0.02
Water intake (ml/100 g b. wt.)	26.50±0.16	25.60±0.11	25.10±0.18	26.90±0.20	25.50±0.21	26.00±0.31

All the values are mean±SE values of six animals in each group

### Body weight

The net body weight gain in all the treated rats was less at both 2 and 4 weeks as compared to the control rats [Table 2]. In accordance with the present observations, Hussain *et al*,[6] have also observed significantly lower body weight gain in cypermethrin-treated rats as compared to control rats. Lakkawar *et al*.[10] observed decrease in body weights in rabbits treated with cypermethrin. Lambda cyhalothrin administered orally to rats has also resulted in reduced body weight gain in both male and female rats.[9]

**Table 2:** Effect of cypermethrin treatment on body weight of treated female albino rats as compared to control rats after 2 and 4 weeks of treatment

Treatment	After 2 weeks			After 4 weeks		
	Initial weight (g/100 g b. wt.)	Final weight (g/100 g b. wt.)	Net body wt. gain (g/100 g b. wt.)	Initial weight (g/100 g b. wt.)	Final weight (g/100 g b. wt.)	Net body wt. gain (g/100 g b. wt.)
Control I	116.60±13.60	140.0±14.70	20.50±0.80	116.60±13.60	160.00±6.23	37.20±0.89
Control II	125.00±0	150.00±2.35	20.00±0.90	125.00±0	167.50±1.76	34.00±0.74
Treated	125.00±0	145.00±2.35	16.00±0.20^a^	150.00±20.40	186.66±27.90	24.40±0.32^a^^b^

a Statistically significantly different (*P* ≤ 0.05) between groups;

b statistically significantly different (*P* ≤ 0.05) between two different time periods; All the values are mean±SE values of six animals in each groups

### Vital organ weight

Weights of all vital organs, viz., liver, spleen, heart, lungs and kidneys were determined on the day of sacrifice and expressed as g/100 g body weight. No significant changes in the weights of vital organs like heart and lungs were observed as compared to their relative control rats after 2 and 4 weeks of treatment. Nagarjuna and Doss[11] have observed pathological changes in hearts of rats orally exposed to cypermethrin for a long duration. Weight of liver and spleen decreased in treated rats at both 2 and 4 wks as compared to the control groups [Table 3], while a significant increase (*P* ≤ 0.05) in the weight of kidneys was observed in cypermethrin-treated rats as compared to control and vehicle treated rats after both 2 and 4 wks of treatment [Table 3]. Liver relative weight also varied nonsignificantly in early stages, but in later stages of the experiment, it significantly decreased in cypermethrin fed rats.[6] Yavasoglu *et al*.[12] have observed no significant change in relative liver weight of cypermethrin-treated rats when compared with control animals at lower dose but at high dose significant decrease in liver weight of treated rats was observed. Some workers have observed dose-dependent increase in liver weight of cypermethrin-treated rats at some acute and subacute dose levels.[2] Adverse changes in liver tissues in test animals have also been observed in cypermethrin-treated male rats as compared to control rats.[13]

**Table 3:** Effect of cypermethrin treatment at 50 mg/kg b. wt. on weights (g/100 g b. wt.) of different organs in control and treated rats after 2 and 4 weeks of treatment

Name of tissue/organ	After 2 weeks			After 4 weeks		
	Control I (g/100 g b. wt.)	Control II (g/100g b. wt.)	Treated (g/100 g b. wt.)	Control I (g/100 g b. wt.)	Control II (g/100 g b. wt.)	Treated (g/100 g b. wt.)
Liver	4.61±0.28	4.62±0.66	4.40±0.01^a^	4.58±0.20	4.82±0.12	4.43±0.07^a^
Spleen	0.24±0.07	0.29±0.04	0.22±0.02^a^	0.26±0.02	0.27±0.07	0.23±0.01^a^
Heart	0.34±0.03	0.40±0.02	0.36±0.03	0.33±0.04	0.36±0.02	0.35±0.06
Lungs	0.34±0.02	0.37±0.05	0.35±0.03	0.32±0.02	0.34±0.01	0.33±0.03
Kidney	0.32±0.01	0.36±0.01	0.39±0.04^a^	0.33±0.08	0.35±0.01	0.40±0.01^a^

a Statistically significantly different (*P* ≤ 0.05) between groups; All the values are mean±SE values of six animals in each group

In the present study, weight of spleen was decreased in the cypermethrin-treated rats after 2 and then further after 4 wks of treatment [Table 3]. The decrease in spleen weight may be indicative of depression of immune status of animals and might reflect a depression of cellular response. Cypermethrin caused increase in kidney weight of treated rats after both 2 and 4 weeks of treatment. Rats exposed to LMR containing allethrin (3.6% w/w) have also shown a significant increase in the weight of kidneys.[2] In male rats, long-term feeding studies with cypermethrin have shown increased kidney weights.[13] Cypermethrin resulted in alteration in the distribution pattern of oxidoreductase in the kidneys of rats. The general loss of oxidoreductases in kidneys of rabbits because of cypermethrin toxicity may be suggestive of decreased metabolism of physiological processes due to degenerative changes in various segments of nephron resulting in concomitant increase of enzymes in the extracellular fluid.[14]

### Endocrine gland weight

Endocrine glands such as adrenal, thyroid and parathyroids were weighed after 2 and 4 weeks of treatment in control and treated rats. Significant increase (*P* ≤ 0.05) in weights of adrenal and thyroid was observed in treated rats at both 2 and 4 weeks as compared to the control rats, and the weight of parathyroid gland decreased significantly in the treated rats at both 2 and 4 weeks as compared to the control rats [Table 4]. Limited data are available regarding endocrine effects in animals following oral exposure to pyrethroids. Significant increase in adrenal weight has been observed in cypermethrin and cybil treated male rats.[15] Increased adrenal weight might reflect a state of physiological stress in the body of rats. Glucocorticoids released from adrenals might play an important role in mediation of depressed immune response. Rats exposed to LMR containing allethrin (3.6% w/w) also had a significant increase in relative weights of adrenal glands in the male rats.[2] In the present study, the weight of the thyroid gland increased in the treated rats at 2 and 4 weeks of treatment. Rats treated with fenvalerate caused a nonsignificant increase in T_3_ and T_4_ concentration.[16] Similarly, increase in thyroid weight has also been observed in rats treated with LMR containing allethrin.[2]

It may be concluded that the changes that were observed in body weight and various organ weights in rats treated with cypermethrin may be indicative of intensity of cellular and tissue damage.
